# Rapid automatic NCS identification using heavy-atom substructures

**DOI:** 10.1107/S0907444902016384

**Published:** 2002-11-26

**Authors:** Thomas C. Terwilliger

**Affiliations:** a Mail Stop M888, Los Alamos National Laboratory, Los Alamos, NM 87545, USA

**Keywords:** non-crystallographic symmetry, heavy-atom substructures

## Abstract

An important component of a fully automated system for structure solution and phase improvement through density modification is a capability for identification of non-crystallographic symmetry as early in the process as possible. Algorithms exist for finding NCS in heavy-atom sites, but currently require of the order of *N* ^5^ comparisons to be made, where *N* is the number of sites to be examined, including crystallographically related locations. A method described here based on considering only sets of sites that have common interatomic distances reduces the computational time by several orders of magnitude. Additionally, searches for proper symmetry allow the identification of NCS in cases where only one heavy atom is present per NCS copy.

## Abstract 2

A rapid algorithm for identifying NCS in heavy-atom sites is described.

## 1. Introduction

Non-crystallographic symmetry (NCS) can be a powerful aid in improving the quality of macromolecular electron-density maps (Rossmann, 1972 ▶; Kleywegt & Read, 1998 ▶). There are many methods for finding NCS (*e.g.* Kleywegt & Read, 1998 ▶; Choi *et al.*, 1997 ▶; Colman *et al.*, 1976 ▶; Bailey *et al.*, 1988 ▶; Lu, 1999 ▶). One particularly useful method for identifying NCS early in the structure-solution process is to search for symmetries in the heavy-atom sites obtained by MAD, SAD or MIR (Buehner *et al.*, 1974 ▶). Recently, Lu (1999 ▶) described an automatic method for identifying symmetry in heavy-atom sites. The method consisted of trying all possible combinations of groups of three sites in an effort to find matching triangles and was demonstrated to be highly effective in finding NCS. The method was rather slow, however, requiring approximately *N* ^5^ comparisons to be examined, where *N* is the number of heavy-atom sites in the region considered for NCS, including all crystallographically related sites.

Here, we describe a related approach for the identification of NCS in heavy-atom sites that is very fast because the only comparisons that are considered are those where interatomic distances in one group at least partially match those in another. Consequently, only a fraction of the possible comparisons need to be made. Additionally, a method using searches for proper symmetry allows the identification of NCS in cases where as few as one heavy atom is present in each NCS copy.

### 1.1. Summary of the method

The basic idea of this method is similar to that of Lu (1999 ▶). Imagine a crystal with six heavy-atom sites. A particular subset of three of these heavy-atom sites (*A*–*B*–*C*) might be NCS-related to another set (*D–E*–*F*) if all the interatomic distances in the first set (*A*—*B*, *A*—*C*, *B*—*C*) match interatomic distances in the second set (*D*—*E*, *D*—*F*, *E*—*F*). The method of Lu (1999 ▶) is to expand the heavy-atom sites using space-group symmetry, then to take all sets of three sites, compare them with all sets of three other sites and find those sets that match in their interatomic distances. These pairs of sets could be related by NCS. If additional sites are present, then they are grouped into existing NCS sets or into new sets with the same interatomic distances if possible. The NCS operators for the crystal are then deduced based on the relationships of these sets. The method works well, but is very slow because of the very large number of comparisons that are required.

The computational requirements of this method can be greatly reduced by noting that a set (*A*–*B*–*E*) cannot possibly be related to a second set (*C*–*D*–*F*) if any one of the three intertomic distances does not match. This means that if *A*—*E* does not match *C*—*F*, we do not have to even consider the distances *A*—*­B*, *B*—*E etc.* Furthermore, it means that the pairs *A*–*E* and *C*–*F*, which have different interatomic distances, never have to be considered as corresponding parts of triplets in combination with any other sites. This vastly reduces the number of comparisons that need to be made.

For example, suppose we have six heavy-atom positions in space group *P*1, with interatomic distances as in Table 1 ▶, and suppose further that we are expecting two sets of three heavy-atom positions related by NCS. Before examining the distances in Table 1 ▶, any pair of atoms (*A*–*E*) might conceivably be NCS-related to any other pair (*e.g. C*–*F*). The distances in Table 1 ▶ limit these possibilities in a very systematic way. The pair *A*–*B*, for example, might be related to the pair *D*–*E* or *D*–*F* (because the interatomic distances are the same), but not to the pairs *C*–*D* or *C*–*E* or *C*–*F* (because the distances are very different).

It is possible to take advantage of the requirement for pairs of distances to match by sorting all pairs of sites according to their interatomic distances and only performing comparisons using pairs of sites that are close together in sequence in the list. In this way, for examples, the pairs *C*–*D*, *C*–*E* and *C*–*F* need never be compared with the pair *A*–*B* because they will be far apart in sequence in this list. This is the key element of the present method.

Continuing with the example in Table 1 ▶, the NCS can be identified in the following steps. Firstly, the pair *A*–*B* is considered as a possible pair of vertices in a triangle representing three sites in one NCS copy. All possible other pairs that could conceivably be the corresponding two vertices in an NCS-related triangle are then listed. These other pairs must share the same interatomic distance. In this case there are only two such possibilities (*D*–*E* and *D*–*F*), both of which have the same interatomic distance as *A*–*B* (20.6 Å). These matching pairs can be obtained without performing comparisons among all pairs because the three pairs *A*–*­B*, *D*–*E* and *D*–*F* will all be very close together in the list of pairs sorted by interatomic distances.

At this point, a reasonable possibility (among many) for Table 1 ▶ is that *A*–*B* corresponds to *D*–*E*. All remaining sites could be considered as possible third vertices in the two triangles. Once again, however, the fact that the interatomic distances must match reduces the number of comparisons that have to be made. In this simple example, there are just two sites (*C* and *F*) that are not yet used, but in another case there might be hundreds. The approach in this case is to note that if site *C* becomes part of the first triangle with *A*–*B* and *F* becomes part of the triangle with *D*–*E*, then the distance *A*—*C* must match the distance *D*—*F*. Accordingly, the pairs *A*–*C* and *D*–*F* must be close in sequence in the list of pairs sorted by interatomic distances and only such pairs of pairs need to be considered. In this example, *A*–*C* and *D*–*F* are both 20.6 Å and this combination is plausible. In the case of the twofold axis considered in Table 1 ▶, the other possibility (*A*–*B*–*F* and *D*–*E*–*C*) is also possible and in fact equally plausible.

## 2. Methods

The core of this method is the sorting of all pairs of sites according to their interatomic distances. The possible pairs of sites that need to be considered can then be limited to those with similar interatomic distances. In general, a set of *m* pairs of sites has the potential for representing *m* NCS copies only if all *m* pairs share (approximately) the same interatomic distance *d*.

The first step is to generate a list of all unique sites crystallographically equivalent to any one of the heavy-atom sites, but as close to the origin as possible. This list is then expanded using crystallographic symmetry to include all sites within a specified distance of the origin, which by default is chosen to be the smallest of the cell translations. This expansion must be over a large enough volume that all the NCS copies are represented at least once.

The second step is to sort all pairs of sites in this list according to their interatomic distances. This is the key step in this procedure; only pairs of sites near to each other in the sequence of this list can be corresponding pairs in different NCS copies.

The third step is to find two or more sets of three sites that have all interatomic distances in common. This step is greatly aided by the sorting of pairs of sites carried out in step 2, because a set of three sites from NCS copy *a* can only be related to three sites from NCS copy *b* if each set of two sites from copy *a* matches a set of two sites from copy *b*. Consequently, it is possible to build up a potential set of three sites in two NCS copies *a* and *b* as follows. Firstly, start with two pairs pair1*a* and pair1*b* of sites that have equal interatomic distances *d*1. Then consider all additional sets of two pairs of sites pair2*a* and pair2*b* with equal interatomic distances *d*2. Finally, consider only the intersection of these two groups where one atom in pair1*a* is the same as one atom in pair2*a* and one atom in pair1*b* is the same as one atom in pair2*b*. In this case, the three atoms in pair1*a* and pair2*a* share all interatomic distances with the three atoms in pair1*b* and pair2*b*. These groups are reasonable candidates for being NCS-related. Additionally, any additional sets of three atoms with the same set of interatomic distances are reasonable candidates for being part of a larger group of NCS-related molecules.

Fourthly, once a group of *m* sets of three atoms is found for which all sets have the same interatomic distances, a set of transformations relating the *m* NCS copies can be identified (provided the interatomic distances are not equal). Any additional atoms that are related to other atoms by these transformations can then be identified and grouped into the corresponding NCS copies.

The fifth step is to refine and score potential NCS solutions. A solution is refined by grouping all the heavy-atom sites into NCS copies (or not including them), then refining the NCS transformations to minimize the r.m.s. deviation among NCS-related sites. The scoring is performed in much the same way as described by Lu (1999 ▶). A set of NCS copies is most likely to be correct if (i) most or all heavy-atom sites are part of an NCS copy and (ii) NCS-related sites are very closely predicted by the NCS transformations. The NCS relationship is particularly likely to be correct if proper NCS is found and if two solutions are found, the one with the higher number of copies is generally more likely to be correct.

Based on these guidelines, two solutions *a* and *b* are compared. Let *N*_NCS,*a*_ and *N*_NCS,*b*_ be the numbers of sites that are part of an NCS copy for solutions *a* and *b* and let *N*_SYM,*a*_ and *N*_SYM,*b*_ be the number of NCS copies for solutions *a* and *b*. If solution *a* has the same or higher symmetry compared with solution *b* (*N*_SYM,*a*_ ≥ *N*_SYM,*b*_) and solution *a* has more sites as part of an NCS copy (*N*_NCS,*a*_ > *N*_NCS,*b*_), solution *a* is always considered better. Also, if solution *a* has lower symmetry (*N*_SYM,*a*_ < *N*_SYM,*b*_), but has many more sites as part of an NCS copy (*N*_NCS,*a*_ *N*_SYM,*a*_ > *N*_NCS,*b*_ *N*_SYM,*b*_), then solution *a* is always considered better.

If all these are equal, then three more quantities are calculated for each solution to help identify which solution is more likely. The first quantity is the r.m.s.d. of the NCS-related sites from positions predicted by NCS (r.m.s.d._NCS,*a*_ and r.m.s.d._NCS,*b*_, for solutions *a* and *b*, respectively). The second is a variable which is 1 if the NCS has point-group symmetry and 0 if not (pg_NCS,*a*_ and pg_NCS,*b*_, for solutions *a* and *b*, respectively). The third is the r.m.s. distance among all the sites in each NCS group (r.m.s._NCS,*a*_ and r.m.s._NCS,*b*_, for solutions *a* and *b*, respectively). Whichever of the two solutions has the lower r.m.s.d. of NCS-related sites from positions predicted by NCS (r.m.s.d._NCS,*a*_ or r.m.s.d._NCS,*b*_) is considered better. If these are equal, then whichever solution has the greater point-group symmetry is considered better. If these are also equal, then whichever solution has the lower r.m.s. distance among all the sites in each NCS group (r.m.s._NCS,*a*_ or r.m.s._NCS,*b*_) is considered better. If all these are also equal, the solutions are considered to be of equal quality.

For cases where fewer than three heavy-atom sites exist per NCS copy, but where proper NCS exists, an alternative approach can be taken. Two general cases can be imagined where NCS can still be deduced. Firstly, NCS can be deduced if there is a twofold axis of symmetry and two sites exist per NCS copy and secondly, NCS can be deduced if there is a threefold or higher axis of symmetry and one or more sites exist per NCS copy.

For the case with a twofold axis and two sites per NCS copy, the sorting of pairs of sites by interatomic distances can again be used to identify potential sets of two pairs of sites that could be related by a twofold axis. Each of these sets of pairs of sets is tested to see whether the four atoms could be related by a twofold axis. This is straightforward because the twofold must pass through two points defined by the mid-points between each potentially twofold-related atom.

For the case with an *N*-fold axis and one site per NCS copy, the sorting of pairs of sites is once again useful because the *N*-fold axis must be made up of a set of *N* atoms, all of which have the same interatomic distance to two other atoms. Consequently, only a very few sets of sites need to be considered at all as potentially *N*-fold related.

In each of these methods, some criterion must be applied to define whether two distances are approximately equal or whether two sites are approximately the same. In practice, a cutoff of about half the resolution of the data is suitable for each of these criteria.

## 3. Results

These approaches for finding NCS in heavy-atom sites were tested using the locations of Se atoms in four data sets containing between nine and 66 sites and containing either proper twofold or threefold symmetry or improper NCS containing up to six copies (Table 2 ▶). The cases tested were a nucleotide diphosphate kinase with nine selenium sites from *Pyrobaculum aerophilum* (Pédelacq *et al.*, 2002 ▶), a hypothetical protein with 16 selenium sites from *P. aerophilum* (J. D. Pedelacq, E. Liong & T. C. Terwilliger, unpublished work), a red fluorescent protein with 26 selenium sites (Yarbrough *et al.*, 2001 ▶) and 2-aminoethylphosphonate transaminase with 66 selenium sites (Chen *et al.*, 2000 ▶). In each case, the sites were those found by running the software *SOLVE* (Terwilliger & Berendzen, 1999 ▶).

In each case the algorithms described above found the known NCS. The CPU time required for finding, sorting, scoring and coming up with a single solution for each case ranged from 1 to 78 s. This compares with 600 to over 10 000 s using the brute-force methods described by Lu (1999 ▶) and implemented in the program *FINDNCS*, using defaults for all parameters or half the cell dimensions as limits for the search region, whichever was successful in the shorter time. In the cases of the 26 sites in red fluorescent protein and the 66 sites of AEP, the *FINDNCS* program was unable to complete the search as a matrix used in calculation was singular.

The approach described here can find NCS relationships in many cases, but does have limitations. For example, some distance cutoff must be used in considering whether two pairs of atoms are likely to be NCS-related, or an infinite number of possibilities would have to be considered. In practice, a cutoff of the smallest of the cell translations works well for this, but in some cases NCS could still be missed. At the other extreme, a cutoff for how similar two distances must be for them to be considered to be NCS-related is also necessary. The cutoff of half the resolution works well in many cases, but might not in cases where heavy-atom sites are not in quite the same places in different molecules. Also, in some cases the scoring system used to choose the NCS may not be optimally weighted. The user has the option to specify the number of NCS copies, however, and this can be used to limit the search to that number.

## 4. Conclusions

The methods described here for rapid identification of NCS in heavy-atom substructures are well suited to being a part of automated structure-solution procedures because they are robust and very quick. They have already proven very useful in automatic NCS symmetry averaging in *RESOLVE* (Terwilliger, 2000 ▶).

## Figures and Tables

**Table 1:** Mock interatomic distances (Å) for six sites in space group *P*1, where sites *A*, *B* and *C* are related by a twofold rotation to sites *C*, *D* and *E*

Sites	*A*	*B*	*C*	*D*	*E*	*F*
*A*	0.0	20.6	20.6	60.0	63.4	63.4
*B*		0.0	10.0	63.4	60.8	60.0
*C*			0.0	63.4	60.0	60.8
*D*				0.0	20.6	20.6
*E*					0.0	10.0
*F*						0.0

**Table 2:** NCS relationships found

Structure	Space group	Sites	NCS	Time (*RESOLVE*) (s)	Time (*FINDNCS*) (s)
NDP kinase	*C*2	9	Threefold	5	624
Hypothetical	*C*2	16	Twofold	8	3700
Red fluorescent protein	*P*2_*1*_	26	4 copies (no point-group symmetry)	12	>9000
2-Aminoethylphosphonate (AEP) transaminase	*P*2_*1*_	66	6 copies (no point-group symmetry)	78	>13000
